# High-density Phenotype Data of Intermediate Phenotypes Associated with Stalk Lodging Resistance in Maize

**DOI:** 10.1038/s41597-025-06008-2

**Published:** 2025-10-30

**Authors:** Bharath Kunduru, Norbert T. Bokros, Kaitlin Tabaracci, Rohit Kumar, Manwinder S. Brar, Christopher J. Stubbs, Yusuf Oduntan, Joseph DeKold, Rebecca Bishop, Joseph Woomer, Virginia Verges, Armando G. McDonald, Christopher S. McMahan, Seth DeBolt, Daniel J. Robertson, Rajandeep S. Sekhon

**Affiliations:** 1 Department of Genetics and Biochemistry, Clemson University, Clemson, SC USA; 2 Department of Horticulture, University of Kentucky, Lexington, KY USA; 3 Center for Quantitative Genetics and Genomics, Aarhus University, Aarhus, Denmark; 4 Department of Mechanical Engineering, University of Idaho, Moscow, ID USA; 5 School of Computer Sciences and Engineering, Fairleigh Dickinson University, Teaneck, NJ USA; 6 Department of Forest, Rangeland and Fire Sciences, University of Idaho, Moscow, ID USA; 7 School of Mathematics and Statistical Sciences, Clemson University, Clemson, SC USA

**Keywords:** Plant breeding, Natural variation in plants

## Abstract

Stalk lodging causes global maize (*Zea mays* L.) yield losses exceeding $6 billion annually. The poorly resolved genetic architecture of stalk lodging resistance, a key determinant of the ability of a plant to remain upright, poses a major constraint for genetic improvement. Characterizing natural variation in plant traits that influence stalk strength across multiple biological scales, referred to as intermediate phenotypes, is critical for enhancing lodging resistance. Here, we present a high-density phenotypic dataset comprising 11 intermediate phenotypes measured on 31,260 stalks from a maize diversity panel of 566 inbred lines grown in four environments. The dataset captures variation in structural and geometric properties of stalks and provides a foundation for genetic mapping, predictive modeling, and machine learning analyses to dissect the genetic basis of stalk lodging resistance. Moreover, trait-level resolution across a genetically diverse panel enables evaluation of the relative contribution of individual phenotypes to stalk strength. Beyond maize improvement for grain and forage production, this dataset offers valuable opportunities for improving stalk lodging resistance in other grasses.

## Background & Summary

Stalk lodging, the permanent displacement of plant stems from their vertical axis, is a major constraint affecting agricultural crop production and one of the most significant sources of potential calorie losses impacting global food security and economic prosperity. In cereals, stalk lodging can reduce grain yield by 2–80% and significantly deteriorate biomass quality^1–3^. Based on global stalk lodging incidence in maize^4,5^, an annual 1% reduction in stalk lodging incidence could feed approximately 5 million more people. Given the substantial economic and food security implications, efforts to enhance stalk lodging resistance, the inherent ability of plant stalks to resist lodging, have focused on both genetic and agronomic approaches. One strategy involves reducing plant height to lower the center of mass, as short plant stature achieved by altering the gibberellin biosynthesis pathway can decrease the stalk lodging incidence^6–8^. Dwarf maize hybrids, suitable for high-density planting, are becoming increasingly favored to mitigate stalk lodging and enhance yield levels^9^. However, high planting densities modify the microenvironment and canopy architecture, leading to increased inter-plant competition, higher shading of leaves, and lower photosynthetic potential, paradoxically exacerbating stalk lodging^10,11^. Breeding for higher grain yields resulted in increased dry matter allocation to the ear, as compared to the stem, and also increased the susceptibility of stalks to lodging in modern maize hybrids^12^. Therefore, comprehensive characterization of the genetic architecture of stalk lodging resistance is critical for minimizing yield losses and improving crop productivity in maize.

Stalk lodging resistance is a complex trait controlled by an interplay between several genetic and environmental factors^13–15^. The poorly resolved genetic basis of this trait is a serious bottleneck to the improvement of stalk strength in crop plants. A major challenge in genetic studies is the lack of a standardized phenotyping method to consistently and reliably quantify stalk lodging resistance^2,16^. Traditionally, field-based phenotypic assessment has relied on late-season lodging incidence determined by counting lodged plants at harvest^17^. However, this method is poorly reproducible due to the heavy influence of various environmental factors, including wind speed, soil moisture, soil fertility, and pest pressure. Another approach that exposes field-grown plants to high-velocity winds also suffers from major drawbacks, including inaccurate phenotypic assessment due to inconsistent wind loading on stems and the high cost of construction and maintenance of the phenotyping equipment. To overcome these limitations, a novel field-based phenotyping platform, Device for Assessing Resistance to Lodging IN Grains (DARLING), was developed to provide measurements of stalk strength by replicating the mechanical loads and stress patterns experienced by naturally lodged plants^18^. Field-based measurements of stalk bending strength and stalk flexural stiffness obtained using the DARLING device were found to be the strongest predictors of stalk lodging incidence across 98 environments spanning four years and 41 unique locations^19^. Among several predictive phenotypes evaluated, including rind penetrometer resistance and cell wall composition traits, stalk bending strength measured by DARLING showed the strongest inverse relationship with lodging, meaning that higher bending strength consistently corresponded to lower lodging incidence in maize. Both stalk bending strength and rind penetrometer resistance are useful for identifying QTLs associated with stalk strength, with each trait capturing partially overlapping but distinct genetic signals related to stalk lodging resistance^20^.

Another major challenge in genetic analysis is that stalk lodging resistance is a complex phenotype that is ultimately determined by structural, material, and geometric properties of stalks manifested at multiple length scales ranging from cell, tissue, organs, and organismal levels^2,21–23^. Identification of these intermediate phenotypes could enable precise genetic analyses by mapping key loci and regulatory networks and facilitate targeted breeding strategies to enhance stalk lodging resistance while minimizing trade-offs. However, the identity of these intermediate phenotypes and their relative contribution to stalk lodging resistance remain poorly resolved. We developed and applied several medium-throughput phenotyping approaches to systematically identify and characterize these intermediate phenotypes^2,16,24,25^. Collectively, these advancements lay the groundwork for detailed phenotypic analysis of multiple traits that regulate stalk lodging resistance and genetic analyses to elucidate the underlying regulatory mechanisms.

Here, we present a comprehensive dataset comprised of 11 phenotypes representing multiple length scales collected on a diversity panel of 566 maize inbred lines evaluated across four environments. These inbred lines represent the major heterotic groups of the North American maize germplasm, stiff stalk, non-stiff stalk, iodent, as well as sweetcorn, popcorn, and tropical lines. The inbred lines evaluated in this dataset have been densely genotyped, enabling future analyses to examine genotype-phenotype relationships using contemporary and novel models. This dataset provides a detailed characterization of the structural and geometric properties of individual internodes located below the primary ear-bearing node. Importantly, the individual plant identity was preserved throughout the study as opposed to averaging phenotypic data across plots. Traditionally, plot averages of multiple plants are used for phenotypic data, but preserving individual plant identities within each inbred of the diversity panel enhances complex trait mapping by capturing phenotypic variability, increasing statistical power, and enabling the implementation of advanced statistical models. This approach also helps disentangle micro-environmental effects within a plot, leading to more accurate estimates of genotypic effects. Additionally, it facilitates multi-trait correlation analysis and improves covariate control, enhancing the detection of rare variants and gene-environment interactions that averaging might obscure.

The stalk phenotype data presented in this dataset is a unique and valuable resource to the plant community for understanding the natural variation in maize stalk architecture, resolving the genetic regulation of stalk lodging resistance, and informing breeding strategies to improve crop productivity and climate resilience. Collecting such comprehensive data is challenging due to the significant cost, labor, and time involved. Generation of this dataset required interdisciplinary expertise to design, implement, and troubleshoot the customized state-of-the-art phenotyping strategies. Given the logistical complexity and scale of effort, we anticipate that the scientific community will find this dataset valuable. This dataset has broad utility in diverse crop improvement applications, including breeding, genetics, Bayesian and frequentist modeling, machine learning, and software development. For example, this dataset was utilized to study the genomic prediction of intermediate phenotypes associated with stalk lodging resistance in maize^26^.

The present dataset is particularly beneficial to research groups working on maize outside North America who may lack access to the germplasm used in this study. This dataset can be combined with phenotype information from other maize germplasm to further enhance the genetic architecture of maize stalk lodging resistance. The inbred lines included in this dataset consist of either entries with expired Plant Variety Protection (ex-PVP) certificates or lines that have been publicly available for several decades. Therefore, the present dataset would be a valuable resource for conducting era studies to compare the dynamics of stalk properties of these historic accessions with recently developed maize germplasm to estimate genetic gains for stalk lodging resistance and related intermediate phenotypes. Moreover, the range of phenotypes included allows researchers to explore the phenotype relationships and trade-offs in breeding maize for bioenergy applications. Beyond biological studies, this dataset also holds engineering relevance for refining stalk geometry and biomechanics, as well as improving phenotyping strategies to enhance stalk lodging resistance in maize.

## Methods

### Plant material and experimental locations

**Fig. 1:**
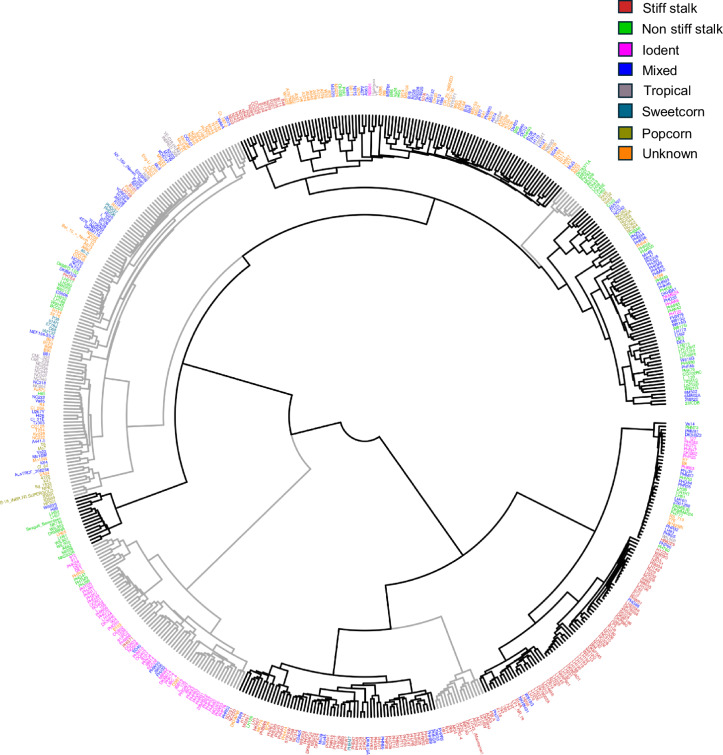
Dendrogram showing phylogenetic relationship among the maize inbred lines evaluated in the present study. The inbred lines representing eight germplasm classes (see legend on top right) are classified into nine clusters as indicated by the black and grey colored branches. Whole-genome resequencing data to construct this dendrogram were obtained from the Dryad database^46^. Only 555 genotypes with available genotype data are shown here.

**Fig. 2:**
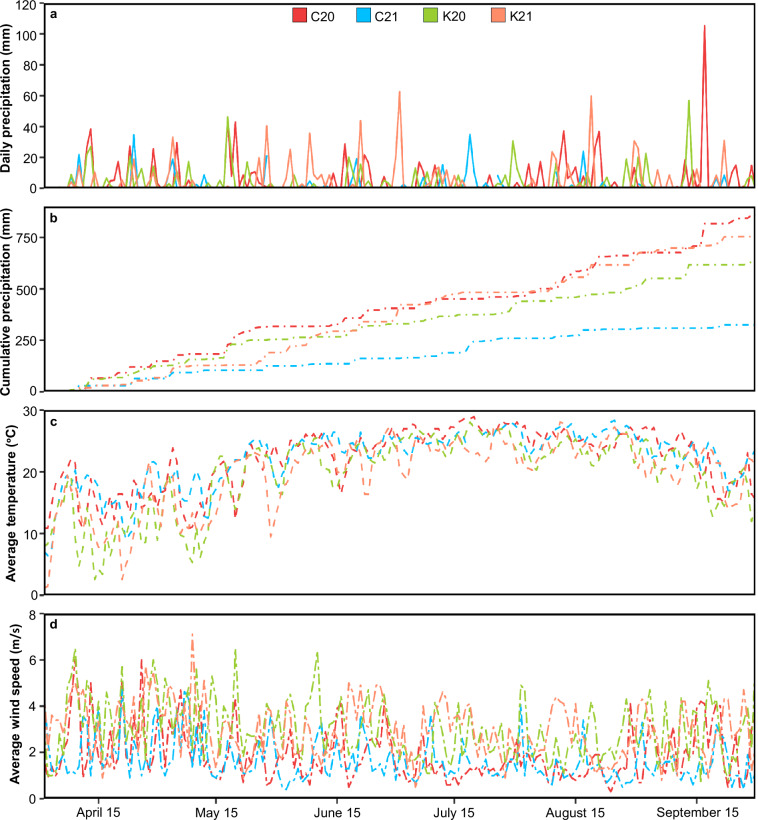
Weather distribution at the experimental locations. The color-codes in the line graphs indicate the experimental locations. (**a**) The solid lines indicate the daily precipitation in experimental locations. (**b**) The dot-dashed lines indicate the cumulative precipitation observed in the experimental locations during the crop growth season. (**c**) The average temperature, calculated as the mean of the maximum and minimum temperatures recorded on each date, is indicated by the dashed lines. (**d**) Average wind speeds at the experimental locations are indicated by the two-dashed lines. C20, Clemson University 2020; C21, Clemson University 2021; K20, University of Kentucky 2020; K21, University of Kentucky 2021; m/s, meters per second.

The present study was conducted on a maize panel consisting of 566 inbred lines, including a subset of the maize Wisconsin diversity panel^27,28^, ex-PVP lines, and other publicly available inbred lines, representing a broad sample of the maize diversity (Fig. 1, Data Table 1). The panel was grown in 2020 and 2021 at the Clemson University Simpson Small Ruminant Research and Education Center, Pendleton, South Carolina (34°37′26.1″N 82°44′11.1″W) and the University of Kentucky Spindletop farm, Lexington, Kentucky (38°07′43.8″N 84°29′19.2″W) (Data Table 2 to Data Table 5). Weather data for the experimental locations, obtained from the National Climatic Data Center database using the Climate Data Online web tool (https://www.ncei.noaa.gov/cdo-web/), indicated diverse environmental conditions at the test locations (Fig. 2, Data Table 6). Due to variable germination and quality control measures, the number of inbred lines studied in each environment varied and ranged between 378–504. In each environment, the experiment was planted in a randomized complete block design with two replications, and each plot consisted of a single 7.62 m row, 0.76 m row spacing, and a total plot area of 5.8 m^2^. At each location, soil tests were conducted to determine the fertilizer application rates and standard agronomic practices were followed to maintain a healthy crop stand in the experimental plots (Table 1). Irrigation and pest control measures were applied in a timely manner to ensure optimal plant health while keeping pest and disease pressure under minimum threshold levels.

**Table 1:** Details of fertilizer application in different environments.

Environment	Basal application	Layby
C20	15-18-23 (NPK) at 446 lbs/acre	32% N at 88 lbs/acre
C21	14-22-22 (NPK) at 350 lbs/acre	32% N at 88 lbs/acre
K20	150 lbs/Acre of N in the form of Urea Ammonium Nitrate	NA
K21	150 lbs/Acre of N in the form of Urea Ammonium Nitrate	NA

N, Nitrogen; P, Phosphorous; K, Potassium;

C20, Clemson University 2020; C21, Clemson University 2021; K20, University of Kentucky 2020; K21, University of Kentucky 2021

### Intermediate phenotypes and sampling strategy

**Fig. 3:**
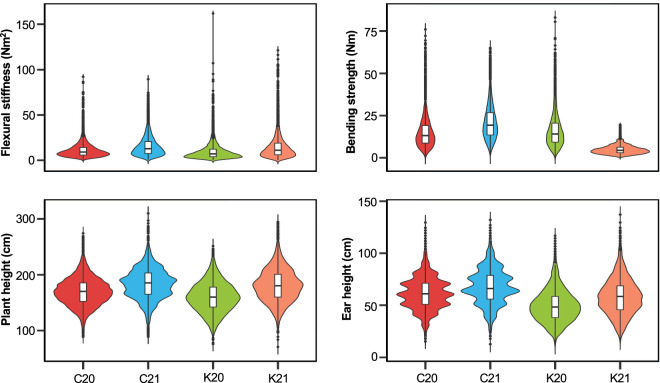
Phenotype distribution of the plant-level intermediate phenotypes associated with stalk lodging resistance. Violin plots represent the distribution of phenotype values in different environments, while the embedded box plots illustrate data distribution around the median. Within each boxplot, the horizontal line represents the median, whereas the lower and upper edges represent the 25^th^ and 75^th^ percentiles, respectively, and the outliers are shown as dots beyond the whiskers. C20, Clemson University 2020; C21, Clemson University 2021; K20, University of Kentucky 2020; K21, University of Kentucky 2021.

**Fig. 4:**
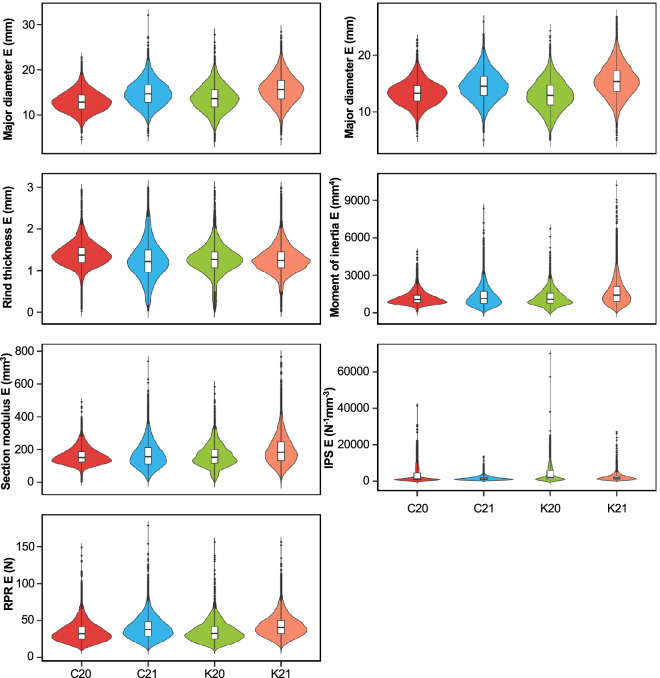
Phenotype distribution of internode-level intermediate phenotypes associated with stalk lodging resistance measured on the internode immediately below the primary ear-bearing node (ear internode). Violin plots represent the distribution of phenotype values in different environments, while the embedded box plots illustrate data distribution around the median. Within each boxplot, the horizontal line represents the median, whereas the lower and upper edges represent the 25^th^ and 75^th^ percentiles, respectively, and the outliers are shown as dots beyond the whiskers. E, Ear internode; IPS, Integrated puncture score; RPR, Rind penetration resistance; C20, Clemson University 2020; C21, Clemson University 2021; K20, University of Kentucky 2020; K21, University of Kentucky 2021.

**Fig. 5:**
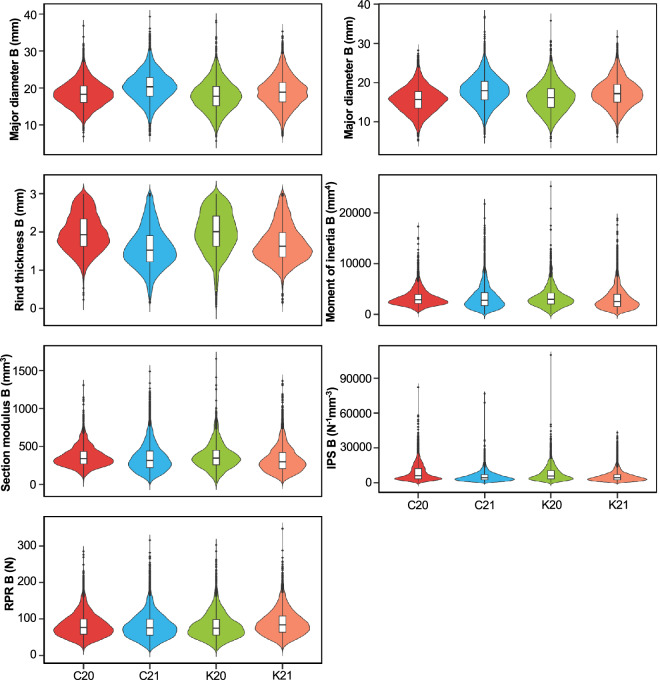
Phenotype distribution of internode-level intermediate phenotypes associated with stalk lodging resistance measured on the first elongated internode above the soil-level (bottom internode). Violin plots represent the distribution of phenotype values in different environments, while the embedded box plots illustrate data distribution around the median. Within each boxplot, the horizontal line represents the median, whereas the lower and upper edges represent the 25^th^ and 75^th^ percentiles, respectively, and the outliers are shown as dots beyond the whiskers. B, Bottom internode; IPS, Integrated puncture score; RPR, Rind penetration resistance; C20, Clemson University 2020; C21, Clemson University 2021; K20, University of Kentucky 2020; K21, University of Kentucky 2021.

To reach the data we aimed to extract from the experiment, the sampling approach needed to consider multiscale measurement with individual plant resolution. Thus, various whole-plant and internode-level phenotypes associated with stalk lodging resistance on individual stalks were recorded while maintaining the stalk identity for each recorded phenotype. Plant-level phenotypes included plant height, ear height, stalk flexural stiffness, and stalk bending strength (Fig. 3). The internode-level phenotypes comprised major diameter, minor diameter, rind thickness, rind penetration resistance, moment of inertia, section modulus, and integrated puncture score experienced by the internodes (Figs. 4–5). The internode-level phenotypes were recorded on all elongated internodes, hereafter referred to as internodes, of the stalk section present below the primary-ear bearing node. Sample preparation, description, and phenotyping protocols for the listed intermediate phenotypes are detailed elsewhere^2,16,29^. Briefly, ten representative and healthy-looking plants per plot were tagged with unique barcode labels to facilitate data matching between field and laboratory measurements. Plant-level phenotypes were collected on live plants in the field, whereas the internode-level phenotypes were measured on the harvested and dried stalks in the laboratory. The phenotyping methodology and relevance of different intermediate phenotypes in assessing stalk lodging resistance are discussed below.

### Days to 50% tasseling

In each plot, during the tasseling growth stage, the number of days from sowing to the initiation of pollen shedding in at least 50% of the plants was recorded^30^.

### Days to 50% silking

In each plot, the number of days from sowing to the initiation of silk emergence in at least 50% of the plants was recorded.

### Plant height

Plant height was recorded one week after days to 50% silking, using polyvinyl chloride pipes marked with metric units, as the linear distance in centimeters (cm) from the tip of the primary rachis of the tassel to the base of the stalk at the ground level. Plant height is a key determinant of stalk lodging resistance, with dwarf genotypes being less susceptible to lodging in various cereal crops, including maize^6,31–33^.

### Ear height

Ear height was recorded as the linear distance in cm from the node bearing the primary ear to the base of the stalk at the ground level. Genotypes with reduced ear height have a lower center of gravity and a shorter lodging arm, both of which enhance stalk lodging resistance^34,35^.

### Stalk flexural stiffness

Stalk flexural stiffness is a strong predictor of stalk lodging resistance and an effective, non-destructive method for assessing stalk strength under field conditions^17^. In the field, stalk flexural stiffness in Newton meter squared (Nm^2^) was measured 38–42 days after 50% silking with DARLING^18,24^ following established protocols^2,16^. For DARLING measurements, the top portions of the plants were removed by excising stalks at the middle of the internode immediately above the primary ear-bearing node. The remaining stalk sections, still rooted in the soil, were prepared by stripping off all lateral appendages to retain bare stalks for measurements. Each prepared stalk was loaded with an incremental force via DARLING until the stalk deflected without breaking or buckling. The reading was discarded if root lodging was observed.

### Stalk bending strength

Stalk bending strength is a reliable predictor of natural stalk lodging incidence, which is used to assess stalk lodging resistance in different crops^19,36^. The methodology for measuring stalk bending strength (a.k.a. maximum bending moment) in Newton meter (Nm) was the same as that for stalk flexural stiffness, except that stalks were loaded with incremental force using DARLING until they buckled or broke. Stalk bending strength is a destructive mode for assessing stalk strength and was therefore recorded after measuring stalk flexural stiffness.

### Major diameter

After phenotyping with DARLING, the stalks were cut at the soil-plant interface and air dried in the greenhouse maintained at 38 °C with a relative humidity of 35%. These dried stalks were used for all the internode-level measurements. Stalk diameter varies around the internode due to the elliptical shape of maize stalks. To account for this variation, two perpendicular diameters, major and minor, were recorded for each internode. Major diameter, measured in millimeters (mm), refers to the largest possible diameter of an internode measured in the direction perpendicular to the plane of the ear groove (if present) or to the leaf attachment point at the basal node, using a Vernier caliper. Stalk diameter is positively associated with stalk lodging resistance in cereals^37,38^.

### Minor diameter

The smallest possible diameter of an internode, measured in mm, was recorded in the direction of the ear groove plane (if present) or the leaf attachment point at the basal node using either a Vernier caliper or an Instron Universal Testing Machine. When measured with the Instron, the probe displacement between the entry and exit points of the stalk tissues was recorded as the minor diameter.

### Rind thickness

In maize stalks, the lack of distinction between rind and pith tissues, which mainly differ in the extent of lignification of parenchyma cell walls^39^, poses challenges for accurately measuring rind thickness. Using the force-displacement curve generated by an Instron Universal Testing Machine during the stalk puncture test, we determined rind thickness as the probe displacement in mm from the rind-pith transition zone to the epidermis^2,25^. As the primary mechanical tissue conferring load-bearing capacity to the stalks, the thickness of the rind plays a crucial role in stalk lodging resistance^40,41^.

### Rind penetration resistance

Rind penetration resistance (a.k.a. rind puncture resistance), measured in Newton (N), was recorded as the peak force from the force-displacement graph obtained by puncturing the stalk tissues with an Instron Universal Testing System. Rind penetration resistance is a widely used parameter to study and improve stalk lodging resistance in maize^42–44^.

### Moment of inertia

Moment of inertia, measured in mm^4^, an indicator of the distribution of cross-sectional material in an elliptic cylindrical maize stalk, was calculated from major diameter, minor diameter, and rind thickness using the formula: ${I=\pi [{qp}}^{3}-(q-2r)(p-{2r)}^{3}]/64$, where *I* denotes the moment of inertia, *p* and *q* represent major and minor diameters, respectively, and *r* indicates the rind thickness. The moment of inertia was reported to be positively associated with stalk lodging resistance and a strong predictor of stalk strength^2,45^.

### Section modulus

Also derived using major diameter, minor diameter, and rind thickness using published protocols and measured in mm^3^. Section modulus has been reported to be a reliable indicator of stalk lodging resistance and not influenced by experimental variables like crop genotype, planting density, etc.^45^. Section modulus is equal to the moment of inertia divided by the minor radius of the stalk cross-section.

### Integrated puncture score

Integrated puncture score is a novel force-displacement weighted measurement of rind penetration resistance developed to increase the efficiency of assessing stalk lodging resistance via rind penetration methodologies. This parameter, measured in N^−1^ mm^−3^, is derived from the force-displacement graph of stalk puncture tests as per published protocols^41^.

**Fig. 6:**
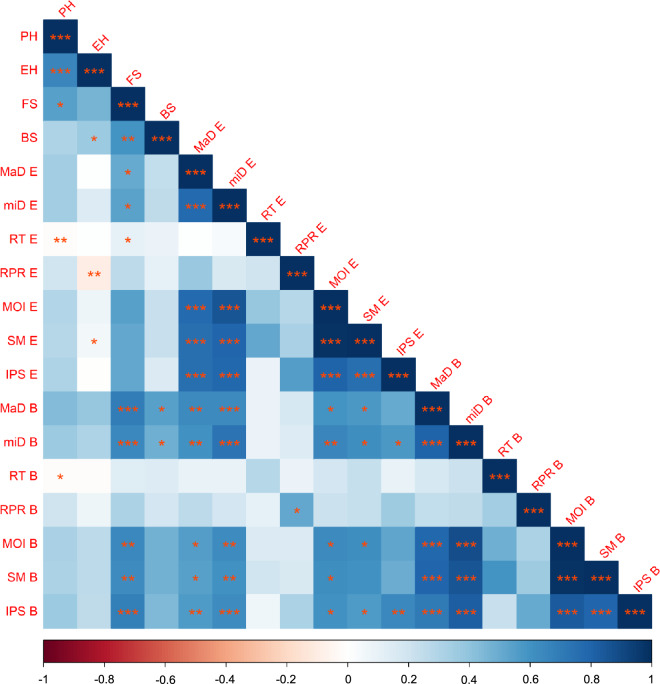
Heatmap of correlation matrix of the intermediate phenotypes associated with stalk lodging resistance. Pearson correlation coefficients are color coded from deep red (-1) to deep blue (1), and the asterisks represent significance levels (*, **, *** correspond to P-value ≤ 0.05, 0.01 and 0.001, respectively). PH, Plant height; EH, Ear height; FS, Stalk flexural stiffness; BS, Stalk bending strength; MaD, Major diameter; miD, Minor diameter; RT, Rind thickness; RPR, Rind penetration resistance; MOI, Moment of inertia; SM, Section modulus; IPS, Integrated puncture score; E, Ear internode; B, Bottom internode.

The summary statistics, including mean, coefficient of variation, and the 5^th^ and 95^th^ percentiles, of the plant- and internode-level phenotypes discussed in the study are presented in Data Table 7. Phenotypic correlations of the intermediate phenotypes are presented in Fig. 6. The range of Pearson correlation coefficients varied from -0.09 to 0.98, which indicated weak to very strong associations between the intermediate phenotypes.

### Naming convention of internodes

In the stalks collected after field phenotyping with DARLING, the number of internodes on the stalk section between the primary-ear bearing node and the base of the stalk at soil level ranged from 4 to 10. To maintain the identity of each internode, we designated these internodes as IN1 to IN10, where IN1 is the internode immediately below the primary ear-bearing node, hereafter referred to as the ear internode in each stalk. Sequentially, the internodes below IN1 were labeled IN2, IN3, and so on, until reaching the bottommost elongated internode, which is labeled as IN*x* and referred to as the bottom internode. For instance, in a stalk with 5 internodes below the primary ear-bearing node, *x* equals 5, and therefore IN5 would be the bottom internode.

### Genotype data

The whole-genome resequencing data for the inbred lines evaluated in the present study was obtained from Dryad^46^ and curated by imposing a stringent low linkage disequilibrium threshold (r^2^ < 0.1), which yielded 1,192,601 biallelic single nucleotide polymorphisms. This curated dataset consisted of weakly correlated single nucleotide polymorphisms, ensuring the inclusion of representative genetic diversity.

## Data Records

All the metadata and phenotype data pertaining to the present study are available at Zenodo^47^. The metadata files include the details of inbred lines studied, weather parameters recorded at the experimental locations, and field maps indicating the planting layouts of the inbred lines evaluated in each environment. Each metadata file is labelled with the prefix “DataTable” followed by a short title describing the contents of the file.

## Technical Validation

Each phenotyping method utilized to collect data has been validated by separate experiments^24,25,41,48–50^. To ensure data uniformity and integrity, we enforced strict quality control measures at every step of phenotyping^16^. In the field, border plants were excluded from data collection to avoid border effects and mechanically damaged, pest-infested, and/or off-type plants were omitted from phenotyping. After data collection, the entire dataset was compiled into a single database and extensively queried to identify errors. Scatter plots of known phenotypic relationships were generated by year, location, inbred line, and as an aggregated data set to search for outliers or irregular trends. For example, flexural stiffness and bending strength are known to be highly correlated intermediate phenotypes^17^, with prior DARLING measurements producing an R^2^ of 0.7–0.85. When plotting aggregate flexural stiffness against bending strength, one location produced a significantly different regression slope, leading to the discovery of a units conversion error. Any outliers were further investigated by reviewing raw data, retaking measurements, or examining the physical stalk specimens for abnormalities. In addition, each phenotype was compared to the ranges observed in prior experiments. For example, rind thickness measurements outside the range of 0.2 to 3.0 mm were flagged for further investigation. The dataset was screened for repeated values, leading to the identification of an instance where the DARLING load cell was maxed out and therefore produced identical values across multiple samples. These quality control measures ensure the accuracy and reliability of the dataset.

## Data Availability

All the data associated with the present study are available at Zenodo^47^.

## Code availability

Scripts and example data for calculating rind penetration resistance, internode diameter, rind thickness, and integrated puncture score are available in published literature^25,41^. Data analyses and figures presented in this manuscript were generated using R Statistical Software https://www.r-project.org (version 4.3.1 for Windows), and the custom R scripts used for making figures are available on figshare^51^.
